# Anticipation modulates neuromechanics of drop jumps in known or unknown ground stiffness

**DOI:** 10.1371/journal.pone.0211276

**Published:** 2019-01-25

**Authors:** Michael Helm, Ramona Ritzmann, Albert Gollhofer, Kathrin Freyler

**Affiliations:** Department of Sport and Sport Science, University of Freiburg, Freiburg i.Br., Germany; Toronto Rehabilitation Institute - UHN, CANADA

## Abstract

With an emphasis on ballistic movements, an accurately anticipated neural control is an essential prerequisite to deliver a motor response coincidentally with the event of ground contact. This study investigated how previous knowledge of the ground condition affects proactive and reactive motor control in drop jumps (DJ). Thereby, human anticipatory capacity of muscle activation was investigated regarding neuromuscular activation, joint kinematics and peak forces associated with DJ performance. In 18 subjects, the effect of knowledge of two different surface conditions during DJs was evaluated. Peak force, ground-contact-time (GCT), rate of force development (RFD) and jump height were assessed. Electromyographic (EMG) activities of the m. soleus (SOL) and gastrocnemius medialis (GM) were assessed for 150ms before (PRE) and during ground contact (GC) for the short-, medium-, and long-latency responses. Ankle and knee joint kinematics were recorded in the sagittal plane.In the unknown conditions peak force, RFD and jump height declined, GCT was prolonged, proactive EMG activity (PRE) in SOL and GM was diminished (P<0.05). During GC, a decline in EMG activity in the unknown condition was manifested for SOL and GM for the SLR, MLR and LLR (P<0.05). Ankle and knee joint deflections during GC were increased in the unknown vs. known condition (P<0.05). Peak force, RFD and jump height were positively correlated to GM-EMG in PRE, SLR, MLR and LLR (P<0.05). Results revealed that proactive and reactive modulations in muscle activity prior and during GC are interrelated to the force-time characteristics and height of the jumps. The unknown condition revealed a comparable neuromuscular activity during pre-activation for both conditions, followed by an inhibition in the subsequent phase after touch down. These findings underline that anticipation is a determining factor influencing timing and adjustment of motor responses to accomplish ballistic movements regarding precision and performance.

## Introduction

Anticipation is one of the key factors which determines human motor performance [1]. Scientific evidence concerning the role of anticipation revealed an impact of major significance on movement accuracy, timing and efficiency [1–4]. Thereby, the temporal and spatial predictability of the stimuli is the most potent determinant of anticipation.

**Fig 1:**
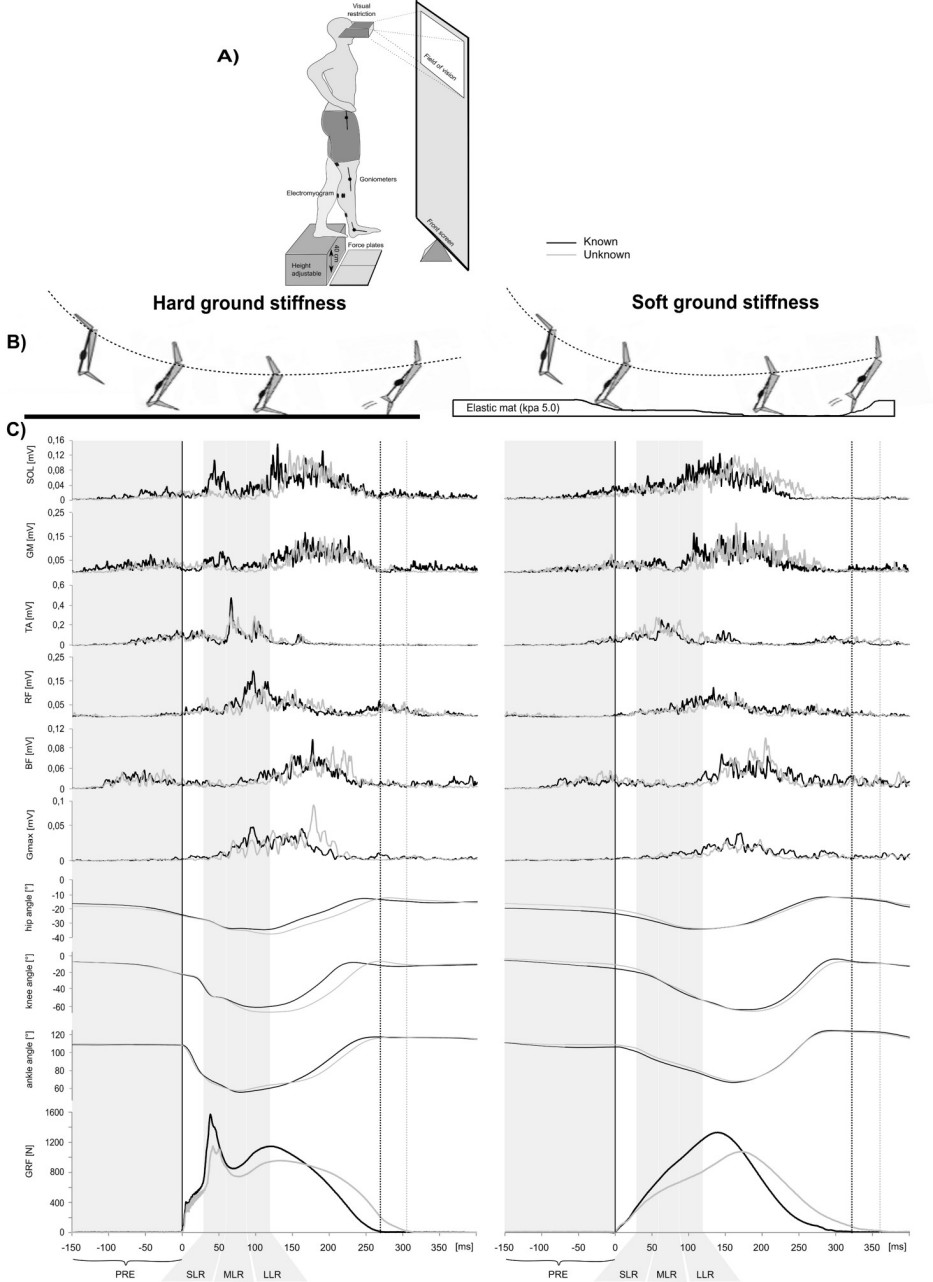
Experimental setting: Schematic illustration of stretch-shortening cycle and force, kinematic and electromyographic activity. **(A)** Experimental setting. **(B)** Schematic illustration of the stretch-shortening cycle with an active stretch (eccentric contraction) of a muscle, followed by immediate shortening (concentric contraction). (**C**) Modulation of the ground reaction force (GRF; bottom), ankle, knee and hip joint kinematics (middle) as well as electromyographic (EMG) activity of shank (SOL, GM and TA) and thigh muscles (RF, BF Gmax) for one representative subject in the known (solid line) and unknown condition (dotted line) on hard ground. GCT is marked as the time between GC and take-off; the relevant EMG phases PRE, SLR, MLR and LLR (bottom) are also marked. Data comprise the means of 15 jumps for each condition. EMG activity in shank and thigh muscles was mostly reduced in PRE, SLR, MLR and LLR from known to unknown. Concomitantly, GCT duration was lengthened, and the peak force decreased from known to unknown conditions.

With an emphasis on ballistic movements, a concisely anticipated neural control is an essential prerequisite to deliver the motor response coincidentally with the event of ground contact. These requirements are based on the natural muscle action underlying ballistic movements, known as the stretch-shortening cycle (SSC). The SSC is defined as the stretching of a pre-activated muscle-tendon complex (Fig 1), immediately followed by muscle shortening in the concentric push-off phase [5–7]. The SSC is associated with an exceedingly high performance due to energy storage in the elastic elements, achieved by the energy transfer from the pre-activated and subsequently eccentrically stretched muscle-tendon complex to the push-off [8,9]. As they are part of regular daily activities (walking, hopping) and human sportive disciplines (jumping, sprinting), the scientific interest in ballistic movements and their adaptability to changes in the environmental conditions has increased in the last decades [7,10–16]. Thereby, the human is interacting with different surface ground stiffnesses (SGS; hard, soft, energy-absorbing or -saving), which needs to be accurately anticipated to produce an adequate performance [11,12,17].

Within conditions were the SGS is known, variation in SGS is associated with changes in peak force [11], jump height [10,11], ground contact time [10,11] and joint kinematics [11,12,17,18]. Concomitantly, adaptations in muscle pre-activation prior to ground contact [11,12,19] and subsequent concentric phase have been reported [11,13]. As manifested in different investigations involving neurophysiological and biomechanical methodologies, the motor pattern is adapted surface-specifically, with leg muscle activation depending on the particular attributes of the SGS [14,20]. This is true for proactive (prior to touch down) and reactive (after touch down) motor strategies, clustered in subdivisions known as the pre-activation, short-, medium- and long-latency responses (SLR, MLR and LLR; [11,13,19]. The SLR, MLR and LLR occur chronologically after touch down and refer to specific control levels within the CNS [21]. Importantly, the level of pre-activation is related to leg stiffness [8] and reduced when performing drop jumps on more elastic compared to stiffer surfaces [11,19]. Additionally, neuromuscular responses after initial ground contact are diminished during jumps on soft surfaces; this diminishment is associated with an increase in joint deflections and a declined jump performance [13]. Authors postulate a mechanically reduced recoil mechanism of the tendo-muscular system through compression, coupled with a modified central control by the nervous system [14] and increased antagonistic co-activation [19] to compensate for the loss of stability at the beginning of the ground contact on softer surfaces [14]. However, what happens when the SGS cannot be anticipated or changes without the jumpers’ awareness? To our knowledge, there are no studies yet which investigated the modulation of the SSC in situations when subjects are facing unknown ground stiffness conditions.

Although the effect of anticipation regarding SGS has not been established yet, and the aforementioned work has been executed without the consideration of anticipation, there is recent evidence that stimulus prediction affects the neuromechanics of ballistic movements. As independently highlighted in experiments using paradigms of differing drop-heights, landing forces or gravitational accelerations [3,4,22–24], unknown compared to known conditions led to a delayed and diminished pre-activation [4,23,24], a reduced neuromuscular activity after GC [4,23,24], concomitant with a massive decline in peak force or jump height [3,4,24] up to a total disruption of the ballistic motor strategy [3]. Thus, to our understanding, it is crucial to know how anticipation modulates the neural control of the leg musculature with an emphasis on the SGSs, and if these modulations are interrelated to the biomechanical output and the individual’s jump performance. Dependencies on anticipation are of high relevance in manifold scenarios of daily life (locomotion on sand, rough terrain in the woods) and athletic disciplines (gymnastics, beach volleyball, cross-country running) as well as for individuals wearing prosthetic limbs exposing them to various surface grounds with differing mechanical attributes that cannot be exactly predicted upfront.

Therefore, this study is to our knowledge the first experiment which compares a known condition with an unknown condition establishing the modulation of the SSC in in regard to anticipation of ground stiffness. The objective of this study was to examine the effects of anticipation on performance variables (e.g., jump height, ground contact time, peak force, rate of force development), joint kinematics and leg muscle activities during drop jumps on hard compared with soft surfaces. Thereby, the SGS was either *known* or *unknown* to the subjects. With an emphasis on neuromechanical coupling, we established the ground reaction force (GRF), joint goniometry and electromyogram (EMG) of relevant muscle groups involved in the SSC as well as interrelations between the muscles’ pre-activation, SLR, MLR and LLR and force output. With reference to the aforementioned findings [4,11,13,19,22], we hypothesised that proactive and reactive changes in leg muscle activities are affected by the anticipation of SGS and are correlated with jump performance. In addition, we expected that differences in leg muscle activities between jumps performed on hard and soft grounds are smaller for the unknown compared to the known condition.

## Materials and methods

### Experimental design

A single-group repeated-measures study design was used to evaluate the effect of surface stiffness anticipation on neuromuscular activity, joint kinematics and force parameters in drop jumps.

### Participants

For this study, 18 subjects (8 females, 10 males, age 24 ± 4.5 years, weight 64 ± 7 kg; height 171 ± 6.9 cm) volunteered. All subjects gave written informed consent to the experimental procedure, which was approved by the ethics committee of the University of Freiburg (Nr. 15/13) and was in accordance with the latest revision of the Declaration of Helsinki. The subjects were healthy with no previous neurological irregularities or injuries of the lower extremity. The sample size was estimated by means of a power analysis based on a previously executed pilot study (test attributes: F-test, repeated-measures analysis of variance, within-between factors, f = 0.25, medium effect; alpha = 0.05; Power = 0.70) [25].

### Testing procedure and paradigm

To evaluate changes in response to anticipation, subjects performed drop jumps from a height-adjustable platform onto the ground in two different situations: The subjects either (1) knew about the ground condition onto which they performed a drop jump or they (2) knew nothing about the ground stiffness. Subjects performed 60 drop jumps from a height of 40 cm [19,26–30], with intermittent breaks of 5 minutes after every 15 jumps to reduce the effects of fatigue. The four jumping conditions consisted of: known hard condition, known soft condition, unknown hard and unknown soft condition, which were described by their characteristics of compression hardness and density. For the hard conditions, jumps were performed on a force plate with a metallic surface (Leonardo, Novotec Medical GmbH, Pforzheim, Germany). For the soft condition, jumps were performed on a mat (10 cm in height, compression hardness of 5 Kpa and a density of 35 kg/m^3^) which laid on the force plate. A loading and unloading compression test of the mat (polyurethane foam) according to DIN EN ISO 3386 was performed by a technical expert, using the Zwick/Roell Z005. The relationship between the applied force and the displacement followed a hysteresis curve. The conditions were performed in a random order.

### Testing preparation

Prior to data collection, subjects performed test jumps for 10 minutes to accustom to the experimental setup and procedures. Subjects jumped barefoot and were instructed to put the hands on the iliac crest. Subjects wore a cap consisting of blinders that restricted their field of view so that only front sight was ensured while performing drop jumps (Fig 1). The platform height was adjusted to account for the depth of mat penetration. Furthermore, subjects were instructed to keep their gaze fixed on a blank screen, placed in 2 m proximity in front of them. In-between each trial, the height-adjustable platform was changed mechanically, arbitrarily going up and down to disorientate the subjects about the platform’s height. This setup prohibited the subject’s ability to cheat and pick up environmental clues that would have falsified the study’s result. Investigators were trained as to how they prepared the next condition; special emphasis was given to reduce any noise level. To preserve a strong test reliability, investigators were given a set of standardised sentences that were mentioned just before the subjects performed the jump.

Known condition: *“**You know* *about the ground stiffness*. *You will perform a drop jump on hard ground/soft ground*. *Get ready to jump–Focus on keeping your legs stiff*, *short GCT and a fast push-off maximally off the ground*.*”*

Unknown condition: *“**You do not know* *the ground stiffness*. *Get ready to jump—focus on keeping your legs stiff*, *short GCT and a fast push-off maximally off the ground*.*”*

Important parameters for excluding a jump for analysis in the known condition were based upon previous studies on drop jumps and SSC behaviour to ensure validity and reliability of the collected data, such as a) ground contact time >350ms for hard and >400ms for soft ground b) knees being greatly flexed at initial ground contact (GC) and during GC or c) subjects either lowered their center of mass or jumping off when leaving the platform before dropping down; both visually observed by a trained operator, d) when the [31] hands were not positioned on iliac crest or e) subjects landed outside the force platform area or two feet landed on the same platform [9,11,18,19,26,27]. Excluding a drop jump from analysis in the unknown condition was based upon different characteristics compared to the known condition, as the drop jumps in unknown conditions are expected to possibly consist of longer ground contact times or greater knee excursions due to the uncertain condition. Drop jumps in the unknown were excluded when the subjects: (a) saw or anticipated the upcoming ground, (b) lowered centre of mass on purpose or jumped off before leaving platform, (c) the hands were not positioned on iliac crest or (d) landed outside the platform area.

Prior to the measurements, subjects performed three isometric maximal voluntary contractions (MVCs) for each recorded muscle, and according to [32] and [33], the trial with the highest EMG was used for data normalisation. The MVCs were executed against resistance for 3 s, with recovery pauses of 1 min between trials and repetitions. Body position during MVCs was strictly controlled and supervised by the authors through goniometric recordings with standardised knee and hip joint angles [34]. Antagonistic muscle activation was monitored, and trials were repeated when antagonists were activated defined as an EMG activity that lasts for more than 25 ms and is 2 standard deviations away from the mean baseline EMG value [35]. Subjects and operators were not blinded, while the assessors were blinded.

### Measurements

#### Force recordings

Vertical GRFs were recorded on a force plate (Advanced Mechanical Technology Inc., Watertown, MA, USA) with a sampling frequency of 2 kHz.

#### Goniometric recordings

Ankle (dorsalflexion and plantarflexion), knee (flexion and extension) and hip (dorsalextension and ventralflexion) joint kinematics in the sagittal plane were recorded by electro-goniometers (Biometrics, Gwent, UK). The ankle goniometer was fixed at the lateral aspect of the right ankle, with its movable endplates attached parallel to the major axis of the foot in line with the fifth metatarsal and the major axis of the leg in line with the fibula. The knee goniometer was placed over the lateral epicondyle of the femur, with one endplate attached to the shank and aligned to the lateral malleolus of the fibula and the other to the thigh aligned to the greater trochanter. For the hip, one endplate was fixed to the lateral pelvis midline and the other to the greater trochanter of the femur. The knee and hip flexion angle were set to zero at 0° during normal upright stance, and joint flexion was reflected by an angle greater than 0°. An angle of 90° between the fifth metatarsal and the fibula corresponded to a 90° ankle angle; an angle greater than 90° reflected plantar flexion. Signals were recorded with a sampling frequency of 2 kHz.

#### Electromyography

Bipolar Ag/AgCl surface electrodes (Ambu Blue Sensor P, Ballerup, Denmark; diameter 9 mm, centre-to-centre distance 34 mm) were placed over the M. soleus (SOL), M. gastrocnemius medialis (GM), M. tibialis anterior (TA), M. rectus femoris (RF) and M. biceps femoris (BF), gluteus maximus (Gmax) of the right leg according to SENIAM [36]. The longitudinal axes of the electrodes were in line with the direction of the underlying muscle fibres. The reference electrode was placed on the tibia. By means of shaving, light abrasion, degreasing, and disinfection of the skin, inter-electrode resistance was kept below 2 kΩ. The EMG signals were transmitted to the amplifier (band-pass filter 10 Hz to 1.3 kHz, 500 x amplified) via shielded cables and recorded with 2 kHz; cables were taped to the skin.

### Data processing

GRFs were used to determine peak force and ground contact time (GCT; time interval between ground contact (GC) and take-off; thresholds for take-offs and landings were set to 3 N). The rate of force development (RFD) values (0–50 ms after GC) were calculated based on reactive index and jump height (H, H = g*t2/8, with t = flight time from the instant of take-off to landing, g = 9.81 m/s2 [37].

For each recorded muscle, the EMG was rectified, averaged, integrated (iEMG) and divided into four time intervals, based on previously reported latencies and durations [38–40]: the pre-activation phase (PRE, 150–0 ms before GC), the SLR (30–60 ms after GC), the MLR (60–85 ms after GC) and the LLR (85–120 ms after GC). Subsequently, iEMGs were time-normalised [mVs] to make them comparable between phases and then normalised to the respective MVC. Furthermore, co-activation of the shank muscles was defined as the ratio of the activation level of TA and the activation level of SOL (TA/SOL), as well as the ratio of the activation level of TA and the activation level of GM (TA/GM). Co-activation of the thigh muscles was defined as the ratio of the activation level of BF and the activation level of RF (BF/RF). Shank and thigh muscle co-activations were calculated for the pre-activation and the SLR, MLR and LLR phases.

The joint angles were determined at GC, and the angular joint excursions (range of motion, ROM) [°] were calculated from GC until the GRF reached its peak.

### Statistics

All statistical analyses were executed using SPSS 23.0 (SPSS Inc., Chicago, Illinois). The effect of anticipation on the variables EMG activity of TA, SOL, GM, RF, BF and Gmax, peak forces, co-activation, joint angles and deflections was evaluated using a two-factor analysis of variance (ANOVA): anticipation x surface stiffness [2, pre vs. post x hard vs. soft]. A priori, the normality of the data was evaluated using the Kolmogorov-Smirnov test; data followed a normal distribution. If the assumption of sphericity, as measured via Mauchly’s test, was violated, the Greenhouse-Geisser correction was used. To correct for multiple testing, the false discovery rate was controlled according to the Benjamini-Hochberg-Yekutieli method, conceptualising the rate of type I errors [41,42]. The level of significance was set to P < 0.05. Effect sizes (partial eta-squared, *η*^2^) were calculated with Cohens *d*. Hereby, an effect size of < 0.01 was interpreted as a small effect, a medium effect size related to an effect size between > 0.01 and < 0.14 and a large effect size agreed to an effect size of > 0.14 [43,44]. Bivariate, two-tailed Pearson´s correlation analyses were conducted to determine the strength of linear relations between the following variables: RFD, peak force, jump height, GCT and PRE EMG for the muscle GM. To establish the interrelationship between proactive and reactive neuromuscular control, bivariate two-tailed Pearson´s correlations were calculated for the variables PRE and SLR of both plantarflexors (SOL and GM).

## Results

No subject dropped out. Fig 1 displays changes in GRFs, EMG activity and joint kinematics in dependency of SGS anticipation in one representative subject. Table 1 contains the grand means of the jump characteristics and joint kinematics. A summary of grand means of the neuromuscular data can be found in Table 2.

**Table 1:** Influence of prediction on forces, jump characteristics and joint kinematics.

*Parameters*	*Surface stiffness HARD*		*Surface stiffness SOFT*		Statistics–rmANOVA (P, F, *η*^2^_p_*)*		
***Forces***	*Known*	*Unknown*	*Known*	*Unknown*	*Main effect Anticipation*	*Main Effect Stiffness*	*Interaction effect*
**Peak force (kN)**	2.1±0.5	1.9±0.5	1.7±0.4	1.6±0.4	**P = 0.001,F(1,17) = 14.7,*η***^**2**^_**p**_ ***=* 0.464**	**P = 0.004,F(1,17) = 11.02,*η***^**2**^_**p**_ **= 0.393**	P = 0.85,F(1,17) = 0.04,*η*^2^_p_ *=* 0.002
**RFD (Nm/s)**	26±15	23±16	12±5	11±5	**P = 0.012,F(1,17) = 67.26,*η***^**2**^_**p**_ **= 0.162**	**P<0.001,F(1,17) = 71.38,*η***^**2**^_**p**_ **= 0.440**	P = 0.46,F(1,17) = 0.57,η^2^_p_ = 0.01
***Jump characteristics***	*Known*	*Unknown*	*Known*	*Unknown*	*Main effect Anticipation*	*Main Effect Stiffness*	*Interaction effect*
**GCT (ms)**	267±72	286±77	330±61	345±67	**P<0.001,F(1,17) = 41.05,*η***^**2**^_**p**_ **= 0.707**	**P<0.001,F(1,17) = 189.02,*η***^**2**^_**p**_ **= 0.917**	P = 0.55,F(1,17) = 0.37,*η*^2^_p_ = 0.021
**Jump height (cm)**	14±5	12±5	12±3	11±3	**P = 0.001,F(1,17) = 16.0,*η***^**2**^_**p**_ **= 0.485**	P = 0.040,F(1,17) = 4.93,*η*^2^_p_ = 0.225	**P = 0.201,F(0,17) = 7.52,*η***^**2**^_**p**_ **= 0.152**
***Kinematics***	*Known*	*Unknown*	*Known*	*Unknown*	*Main effect Anticipation*	*Main Effect Stiffness*	*Interaction effect*
**Hip joint**							
**Angle at GC (°)**	13±6	11±8	12±7	10±6	**P = 0.049,F(1.17) = 4.49,*η***^**2**^_**p**_ **= 0.209**	P = 0.929,F(1,17) = 0.008,*η*^2^_p_ = 0.000	**P<0.001,F(1,17) = 24.05,*η***^**2**^_**p**_ **= 0.586**
**Amplitude during GC (Δ°)**	16±7	18±7	12±7	15±6	**P<0.001,F(1,17) = 22.0,*η***^**2**^_**p**_ **= 0.565**	**P = 0.001,F(1,17) = 17.4,*η***^**2**^_**p**_ **= 0.506**	P = 0.54,F(1.17) = 0.39,*η*^2^_p_ = 0.022
**Knee joint**							
**Angle at GC (°)**	21±4	16±5	18±5	15±4	**P<0.001,F(1,17) = 31.1,*η***^**2**^_**p**_ **= 0.69**	**P<0.001,F(1,17) = 71.04,*η***^**2**^_**p**_ **= 0.835**	**P = 0.006,F(1,17) = 10.38,*η***^**2**^_**p**_ **= 0.426**
**Amplitude during GC (Δ°)**	39±11	42±11	33±11	38±10	**P<0.001,F(1,17) = 38.4,*η***^**2**^_**p**_ **= 0.694**	**P<0.001,F(1,17) = 19.36,*η***^**2**^_**p**_ **= 0.532**	P = 0.09,F(1,17) = 3.16,***η***^**2**^_p_ = 0.16
**Ankle joint**							
**Angle at GC (°)**	117±7	116±7	112±7	115±7	**P<0.001,F(1,17) = 25.69,*η***^**2**^_**p**_ **= 0.602**	**P = 0.001,F(1,17) = 15.92,*η***^**2**^_**p**_ **= 0.484**	**P<0.001,F(1,17) = 49.64,*η***^**2**^_**p**_ **= 0.745**
**Amplitude during GC (Δ°)**	54±8	54±8	38±9	40±10	P = 0.059,F(1,17) = 4.13,*η*^2^_p_ = 0.205	**P<0.001,F(1,17), = 78.49,*η***^**2**^_**p**_ **= 0.831**	**P = 0.03,F(1.17) = 5.72,*η***^**2**^_**p**_ **= 0.263**

Changes in peak force and RFD are illustrated at the *top*. Adaptations in GCT and jump height are displayed in the *middle*. Ankle, knee and hip joint kinematics at initial and during GC are displayed at the *bottom*. Values represent means ± SD; P and F values indicate significant changes.

**Table 2:** Changes in electromyographic activity in shank and thigh muscles as well as co-activation ratio for thigh muscles.

*Parameters*	*Surface stiffness HARD*		*Surface stiffness SOFT*		Statistics—rmANOVA (P, F, *η*^2^_p_*)*		
***EMG PRE***	*Known*	*Unknown*	*Known*	*Unknown*	*Main effect Anticipation*	*Main Effect Stiffness*	*Interaction effect*
**SOL**	0.58±0.15	0.53±0.15	0.49±0.13	0.52±0.13	**P = 0.03,F(1,17) = 7.86,*η***^**2**^_**p**_ **= 0.273**	**P<0.001,F(1,17) = 19.62,*η***^**2**^_p_ **= 0.536**	**P = 0.002,F(1,17) = 13.72**,***η***^**2**^_**p**_ **= 0.447**
**GM**	0.91±0.46	0.84±0.41	0.81±0.36	0.84±0.40	**P = 0.02,F(1,17) = 7.07,*η***^**2**^_**p**_ **= 0.194**	P = 0.294,F(1,17) = 1.17,*η*^2^_p_ = 0.064	**P = 0.007,F(1,17) = 10.25,*η***^**2**^_**p**_ **= 0.360**
**TA**	0.51±0.17	0.49±0.15	0.54±0.17	0.52±0.17	**P = 0.004,F(1,17) = 10.615,*η***^**2**^_**p**_ **= 0.37**	P = 0.809,F(1,17) = 0.061,*η*^2^_p_ = 0.004	P = 0.50,F(1,17) = 0.48,*η*^2^_p_ = 0.029
**RF**	0.51 ±0.22	0.49±0.21	0.47±0.22	0.49±0.22	**P = 0.02,F(1,17) = 6.2,*η***^**2**^_**p**_ **= 0.267**	**P<0.001,F(1,17) = 50.33,*η***^**2**^_**p**_ **= 0.748**	P = 0.81,F(1,17) = 0.06,*η*^2^_p_ = 0.003
**BF**	0.32±0.15	0.34±0.15	0.31±0.15	0.34±0.15	**P = 0.001,F(1,17) = 45.92,*η***^**2**^_**p**_ **= 0.558**	P = 0.05,F(1,17) = 4.66,*η*^2^_p_ = 0.215	**P = 0.002,F(1,17) = 25.98**,***η***^**2**^_**p**_ **= 0.460**
**Gmax**	0.30±0.34	0.30±0.24	0.28±0.34	0.28±0.34	P = 0.28,F(1,17) = 1.27,*η*^2^_p_ = 0.070	P = 0.66,F(1,17) = 2.01,*η*^2^_p_ = 0.012	P = 0.58,F(1,17) = 0.30,*η*^2^_p_ = 0.019
**BF/RF**	74±30	72±31	75±32	75±31	P = 0.27,F(1,17) = 1.34,*η*^2^_p_ = 0.09	P = 0.06,F(1,17) = 4.21,*η*^2^_p_ = 0.23	P = 0.32,F(1,17) = 1.08,*η*^2^_p_ = 0.72
***EMG SLR***	*Known*	*Unknown*	*Known*	*Unknown*	*Main effect Anticipation*	*Main Effect Stiffness*	*Interaction effect*
**SOL**	1.02±0.43	1.01±0.47	0.74±0.26	0.63±0.22	**P = 0.03,F(1,17) = 5.64**,***η***^**2**^_**p**_ **= 0.249**	**P = 0.002,F(1,17) = 13.38,*η***^**2**^_**p**_ **= 0.44**	P = 0.08,F(1,17) = 3.42,*η*^2^_p_ = 0.168
**GM**	0.86±0.36	0.79±0.31	0.76±0.31	0.69±0.27	**P = 0.01,F(1,17) = 8.73**,***η***^**2**^_**p**_ **= 0.339**	**P = 0.014,F(1,17) = 7.47,*η***^**2**^_**p**_ **= 0.014**	P = 0.71,F(1,17) = 0.15,*η*^2^_p_ = 0.009
**TA**	0.53±0.20	0.55±0.20	0.59±0.22	0.59±0.21	P = 0.48,F(1,17) = 0.53,*η*^2^_p_ = 0.032	P = 0.124,F(1,17) = 2.63,*η*^2^_p_ = 0.141	P = 0.72,F(1,17) = 0.13,*η*^2^_p_ = 0.008
**RF**	1.33±0.82	1.26±0.82	1.03±0.82	0.93±0.81	**P = 0.005,F(1,17) = 10.30**,***η***^**2**^_**p**_ **= 0.377**	**P<0.001,F(1,17) = 19.45,*η***^**2**^_**p**_ **= 0.534**	P = 0.41,F(1,17) = 0.70,*η*^2^_p_ = 0.040
**BF**	0.37±0.19	0.33±0.17	0.30±0.14	0.30±0.14	**P = 0.03,F(1,17) = 5.59**,***η***^**2**^_**p**_ **= 0.248**	**P = 0.012,F(1,17) = 8.00,*η***^**2**^_**p**_ **= 3.20**	P = 0.16,F(1,17) = 2.18,*η*^2^_p_ = 0.113
**Gmax**	0.67±0.43	0.58±0.43	0.47±0.34	0.47±0.37	**P = 0.01,F(1,17) = 7.56, *η***^**2**^_**p**_ **= 0.308**	**P = 0.006,F(1,17) = 9.83,*η***^**2**^_**p**_ **= 0.366**	**P = 0.02,F(1,17) = 7.30**,***η***^**2**^_**p**_ **= 0.300**
**BF/RF**	35±17	34±17	41±24	46±26	P = 0.1,F(1,17) = 3.0,*η*^2^_p_ = 0.16	**P = 0.006,F(1,17) = 9.81,*η***^**2**^_**p**_ **= 0.05**	P = 0.05,F(1,17) = 4.5,*η*^2^_p_ = 0.22
***EMG MLR***	*Known*	*Unknown*	*Known*	*Unknown*	*Main effect Anticipation*	*Main Effect Stiffness*	*Interaction effect*
**SOL**	1.24±0.62	1.09±0.54	0.79±0.28	0.66±0.25	**P<0.001,F(1,17) = 28.19,*η***^**2**^_**p**_ **= 0.653**	**P = 0.004,F(1,17) = 11.22,*η***^**2**^_**p**_ **= 0.428**	P = 0.95,F(1,17) = 0.01,*η*^2^_p_<0.001
**GM**	0.95±0.50	0.80±0.42	0.69±0.34	0.60±0.28	**P = 0.002,F(1,17) = 13.29,*η***^**2**^_**p**_ **= 0.439**	**P<0.001,F(1,17) = 19.45,*η***^**2**^_**p**_ **= 0.534**	P = 0.15,F(1,17) = 2.25,*η*^2^_p_ = 0.117
**TA**	0.65±0.32	0.71±0.38	0.73±0.37	0.72±0.31	P = 0.12,F(1,17) = 2.70,*η*^2^_p_ = 0.144	P = 0.539,F(1,17) = 0.395,*η*^2^_p_ = 0.024	P = 0.15,F(1,17) = 2.24,*η*^2^_p_ = 0.123
**RF**	1.35±0.89	1.27±0.86	1.21±0.83	1.16±0.86	P = 0.06,F(1,17) = 3.99,*η*^2^_p_ = 0.190	P = 0.148,F(1,17) = 2.3,*η*^2^_p_ = 0.119	P = 0.06,F(1,17) = 4.14,*η*^2^_p_ = 0.196
**BF**	0.71±0.36	0.69±0.36	0.65±0.34	0.61±0.36	P = 0.25,F(1,17) = 1.44,*η*^2^_p_ = 0.078	P = 0.054,F(1,17) = 4.3,*η*^2^_p_ = 0.202	P = 0.75,F(1,17) = 0.11,*η*^2^_p_ = 0.006
**Gmax**	0.90±0.48	0.77±0.46	0.59±0.42	0.57±0.41	**P = 0.003,F(1,17) = 11.63,*η***^**2**^_**p**_ **= 0.406**	**P = 0.001,F(1,17) = 16.83,*η***^**2**^_**p**_ **= 0.497**	**P = 0.004,F(1,17) = 11.23,*η***^**2**^_**p**_ **= 0.398**
**BF/RF**	63±34	67±35	69±45	67±42	P = 0.77,F(1,17) = 0.092,*η*^2^_p_ = 0.005	P = 0.43,F(1,17) = 0.64,*η*^2^_p_ = 0.036	P = 0.36,F(1,17) = 0.90,*η*^2^_p_ = 0.05
***EMG LLR***	*Known*	*Unknown*	*Known*	*Unknown*	*Main effect Anticipation*	*Main Effect Stiffness*	*Interaction effect*
**SOL**	1.22±0.79	1.05±0.57	1.16±0.54	0.97±0.37	**P = 0.006,F(1,17) = 9.82,*η***^**2**^_**p**_ **= 0.366**	P = 0.529,F(1,17) = 0.413,*η*^2^_p_ = 0.24	P = 0.81,F(1,17) = 0.06,*η*^2^_p_ = 0.004
**GM**	1.15±0.69	0.98±0.51	1.25±0.70	1.12±0.65	**P = 0.001,F(1,17) = 15.44,*η***^**2**^_**p**_ **= 0.476**	P = 0.066,F(1,17) = 3.85,*η*^2^_p_ = 0.185	P = 0.66,F(1,17) = 0.20,*η*^2^_p_ = 0.012
**TA**	0.57±0.26	0.59±0.22	0.63±0.31	0.64±0.27	P = 0.66,F(1,17) = 0.20,*η*^2^_p_ = 0.013	P = 0.090,F(1,17) = 3.26,*η*^2^_p_ = 0.169	P = 0.82,F(1,17) = 0.05,*η*^2^_p_ = 0.003
**RF**	1.35±0.82	1.30±0.92	1.46±1.0	1.35±0.92	P = 0.25,F = (1,17) = 1.44,*η*^2^_p_ = 0.078	P = 0.361,F(1,17) = 0.882,*η*^2^_p_ = 0.049	P = 0.36,F(1,17) = 0.89,*η*^2^_p_ = 0.050
**BF**	0.51±0.26	0.49±0.26	0.46±0.24	0.44±0.26	P = 0.25,F(1,17) = 1.43,*η*^2^_p_ = 0.078	P = 0.054,F(1,17) = 4.29,*η*^2^_p_ = 0.202	P = 0.75,F(1,17) = 0.11,*η*^2^_p_ = 0.006
**Gmax**	0.95±0.50	0.88±0.54	0.71±0.33	0.63±0.37	**P = 0.008,F(1,17) = 9.15,*η***^**2**^_**p**_ **= 0.350**	**P = 0.002,F(1,17) = 12.73,*η***^**2**^_**p**_ **= 0.428**	P = 0.88,F(1,17) = 0.02,*η*^2^_p_ = 0.001
**BF/RF**	47±24	46±23	42±30	42±28	P = 0.96,F(1,17) = 0.002*η*^2^_p_ = 0.000	P = 0.4,F(1,17) = 0.75,*η*^2^_p_ = 0.04	P = 0.61,F(1,17) = 0.27,*η*^2^_p_ = 0.02

Data were normalised to iEMG during maximal voluntary contraction (EMG MVC) in regard to anticipation in the relevant phases: PRE (150 ms before GC until touchdown), SLR (30–60 ms after GC), MLR (60–90 ms after GC) and LLR (90–120 ms after GC). Values were normalised to EMG during MVC and are expressed as means ± SD. Co-activation ratios of m. biceps femoris and m. rectus femoris (BF/RF) are also expressed for the respective phases.

### Forces and jump performance

**Fig 2:**
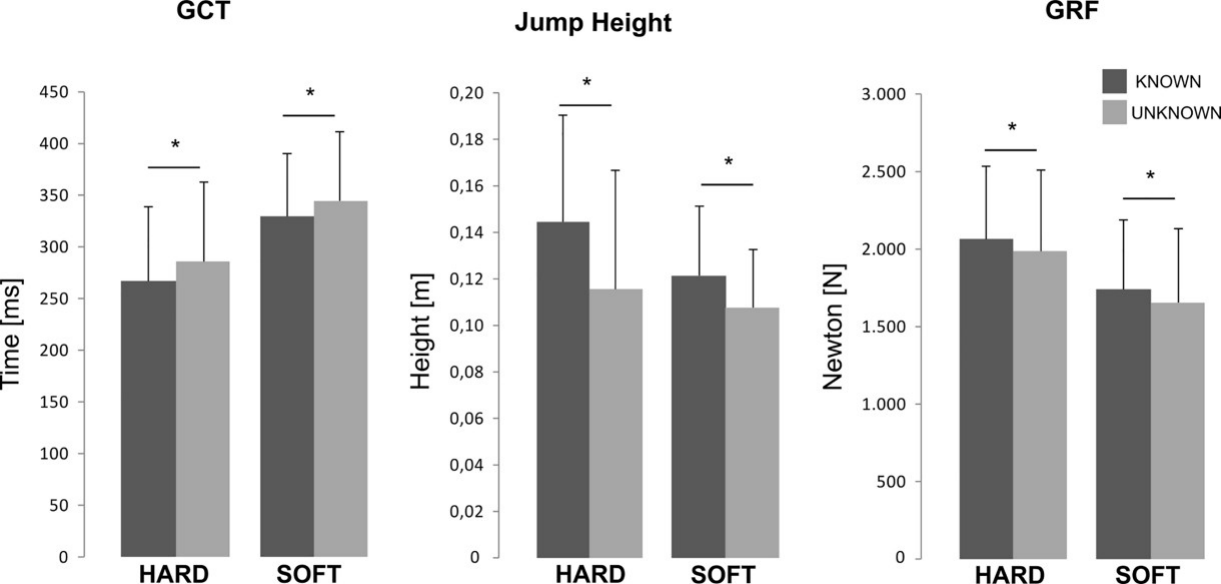
Differences in GCT, jumping height and GRF in known and unknown conditions. Differences in GRFs, GCTs and jumping heights between unknown and known conditions are shown in respect to the surface ground stiffness (soft and hard). Dark columns display the results of the known condition, light grey columns those of the unknown condition. Significant results are marked with an asterisk (* for P < 0.05).

Unknown in contrast to known conditions induced a significant decrease in peak force, RFD and jumping height (Table 1 and Fig 2). Concomitantly, GCT increased. When the SGS was known, soft compared to hard ground stiffness caused a decline in peak force, RFD and jumping height, whereas GCT increased.

### Joint kinematics

Ankle, knee and hip joint angles at initial touchdown as well as ROM during GCT changed with anticipation, as manifested by significant main and interaction effects (Table 1). At initial touch down, no differences in ankle, knee and hip joint angles were found for the unknown conditions between soft and hard surfaces (Fig 1), whereas distinct changes were manifested for the known conditions: the ankle joint was more dorsiflexed, and the knee and hip joints were more extended at initial touchdown in the jumps performed on the soft compared to the hard surface (Table 1). This might indicate an effect of anticipation, as reflected in a pre-adjustment according to the upcoming surface condition. During GC, the ROM was increased in the knee and hip joint in the unknown compared to the known condition. The ankle ROM remained unaffected (Table 1).

### Electromyographic activity

We found proactive and reactive dependencies of the leg muscles’ EMG activity on anticipation. Prior to GC, unknown in contrast to known conditions caused changes in EMG activity in SOL, GM, TA, RF and BF (Table 2). When the SGS was known, EMG activity during pre-activation was significantly diminished for the jumps performed on soft compared to hard ground. In contrast, when the SGS was unknown, there was no differentiation between the soft and hard surface, displaying a comparable amount of pre-activation for both ground stiffness conditions.

During ground contact, a decline in EMG activity was manifested for the phases during GC in most of the skeletal muscles in the unknown in contrast to the known conditions as follows: during SLR for SOL, GM, RF, BF and Gmax. For MLR and LLR, a significant reduction was manifested for SOL, GM and Gmax.

### Co-activation

**Fig 3:**
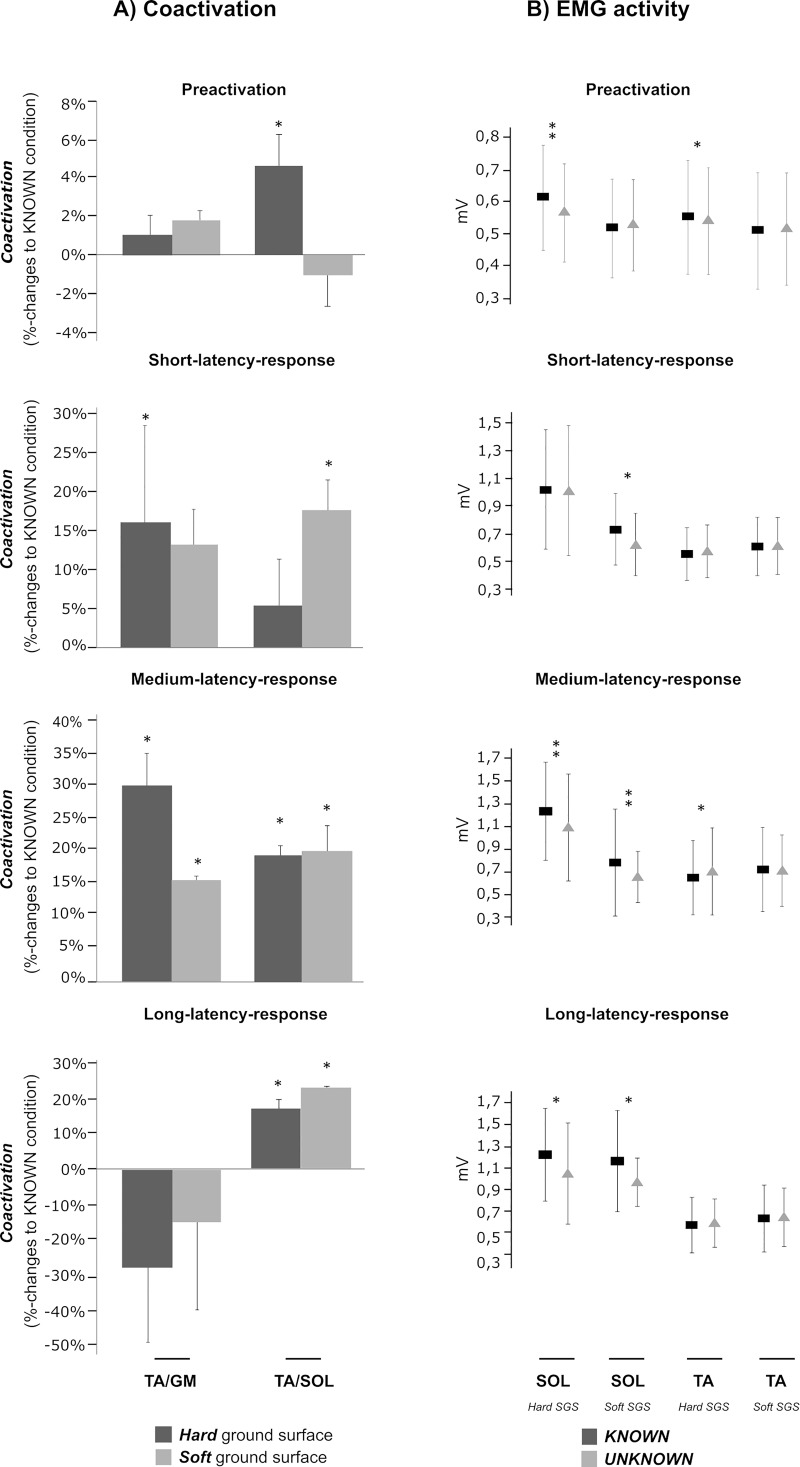
Comparison between changes in antagonistic co-activation in the shank musculature to measured EMG activity. **(A)** Percentile changes in antagonistic co-activation in the shank musculature, expressed as percentage changes to the known conditions for the ratios m. tibialis anterior and m. soleus (TA/SOL) and m. gastrocnemius medialis (TA/GM), respectively (hard ground displayed in dark columns, soft ground displayed in lighter grey columns). From top to bottom, the graphs demonstrate the results for: PRE (-150-0 ms prior to ground contact), SLR (30–60 ms), MLR (60–90 ms) and LLR (90–120 ms) phases. Significant results are marked with an asterisk (* for P < 0.05; **for P < 0.001). **(B)** Changes in grand means in SOL and TA are expressed as differences between known and unknown conditions in respect to the SGS (hard and soft). The dark squares display the grand means with standard deviations of the known condition; the triangles display the grand means of the unknown condition. From top to bottom, the graphs demonstrate the results for: PRE, SLR, MLR and LLR phases. Significant results are marked with an asterisk (* for P < 0.05; ** for P < 0.001).

Changes in shank co-activation are illustrated in Fig 3, changes in thigh muscle co-activation (BF/RF) displayed in Table 2. Antagonistic pairs of shank (TA/SOL and TA/GM) muscles demonstrated a significantly increased level of co-activation in the unknown compared to the known condition: For hard ground stiffness, TA/SOL and TA/GM showed a significantly greater co-activation in the unknown compared to the known condition in PRE, while no differences were observed for soft ground. Furthermore, TA/SOL co-activation was increased in SLR and MLR for both SGSs in the unknown condition. Likewise, TA/SOL and TA/GM were increased in LLR in the unknown condition, independent of the SGS. For BF/RF co-activation, results showed no significant effect of anticipation.

### Correlations

**Fig 4:**
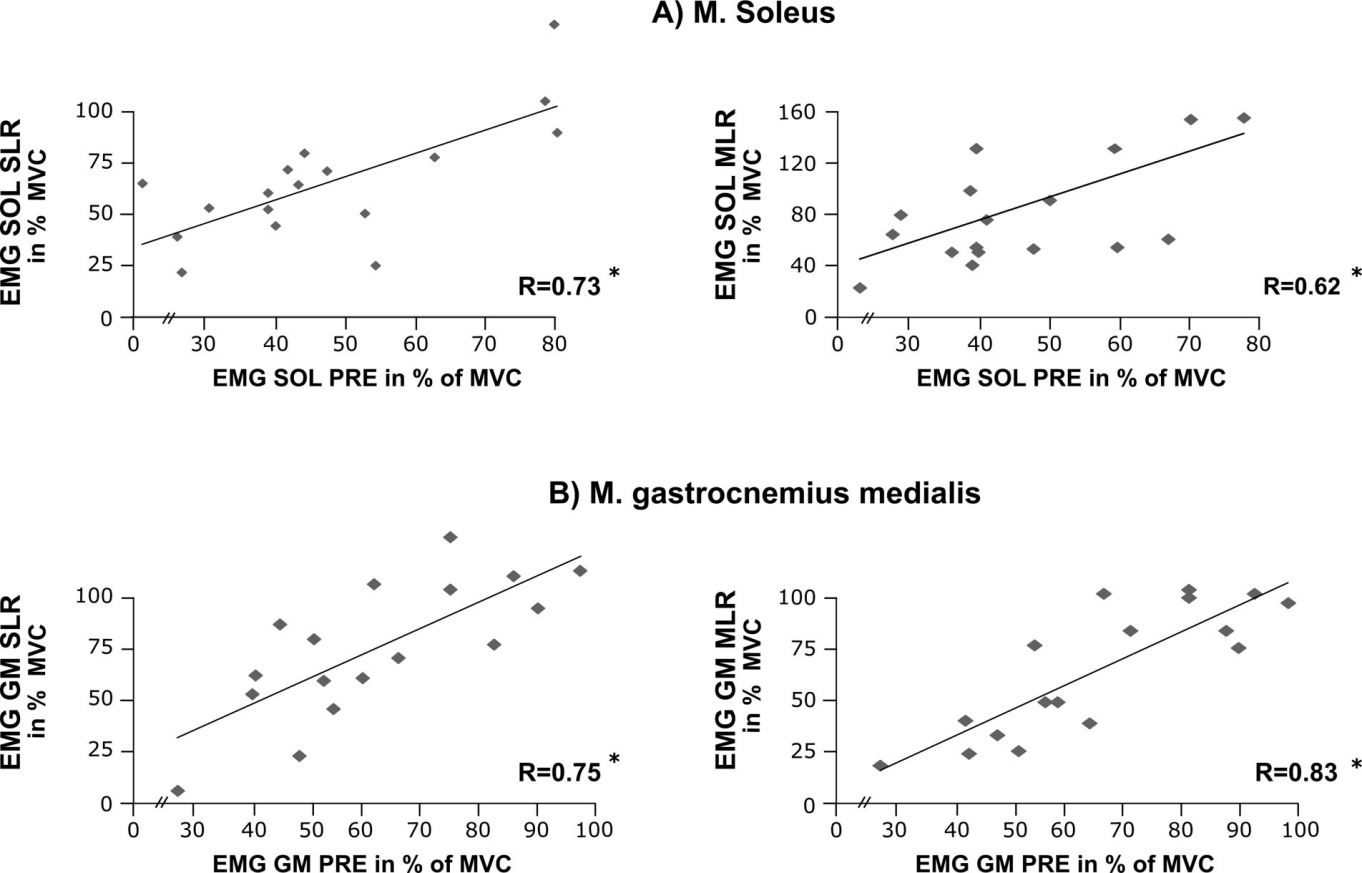
Proactive and reactive bivariate correlations and correlation coefficients of shank muscles. Correlations among the variables of EMG pre-activation (PRE, abscissas), short-latency response (SLR, left) or medium-latency response (MLR, right), respectively, for the shank muscles **(A)** M. soleus and **(B)** M. gastrocnemius medialis (ordinate). Results are illustrated for the drop jumps performed in the unknown condition on the hard surface. These findings illustrate the dependency of the dorsiflexors’ activation intensity after touch-known on the muscles’ pre-activity prior to touch-down. * Significant findings (P < 0.05).

**Fig 5:**
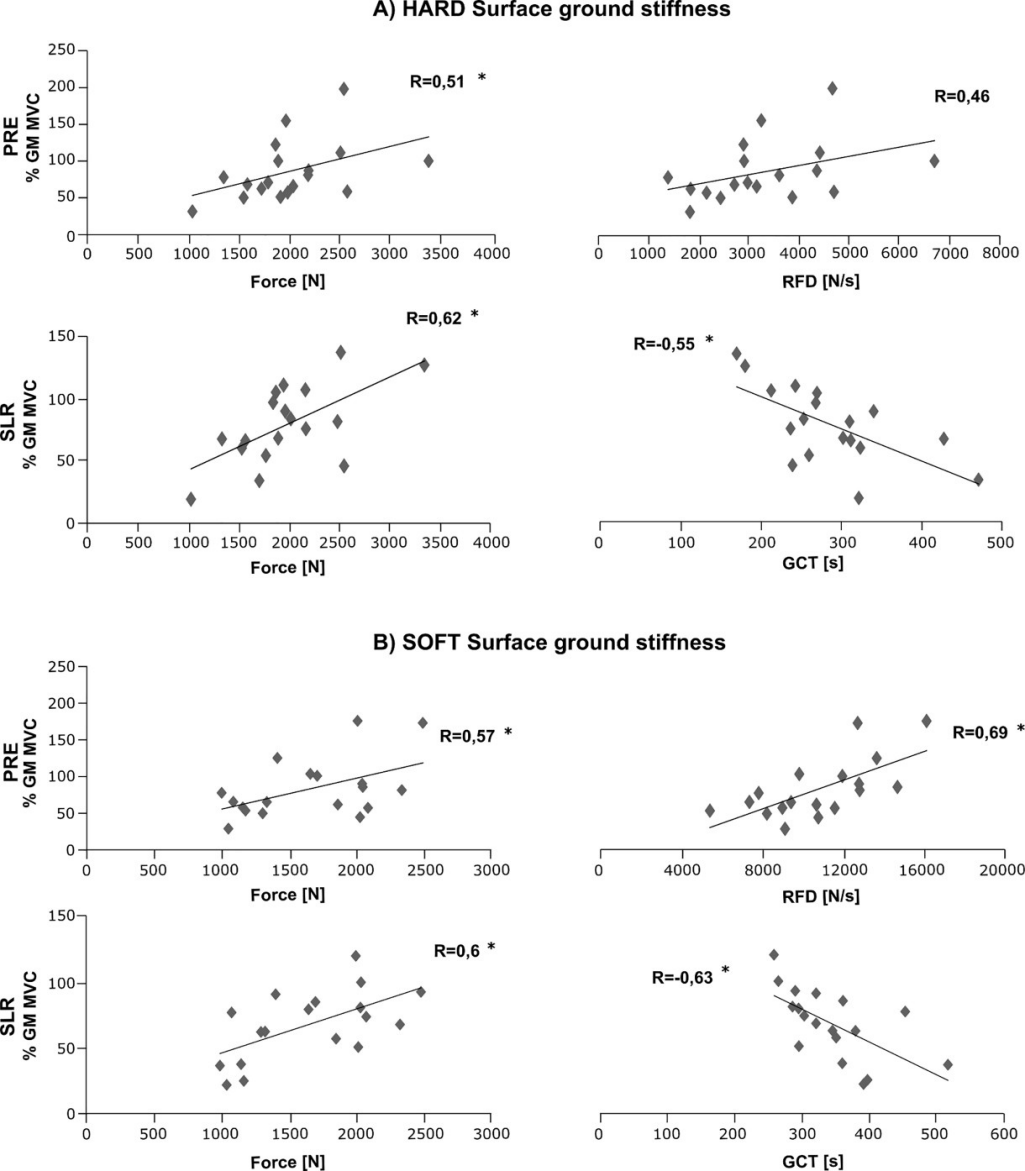
Bivariate correlations and coefficients among the variables peak force, RFD and GCT. Normalised EMGs of the M. gastrocnemius medialis in the relevant EMG phases on the ordinate for the drop jumps performed in the unknown condition in the pre-activation phase (PRE: -150–0 ms before ground contact until ground contact) and the short-latency response (SLR: 30–60 ms after ground contact). For both (A) hard and (B) soft surface ground stiffness, the strength of the relationship between force-time characteristics and the proactive and reactive neuromuscular set was moderate to high. * Significant findings (P < 0.05).

Corresponding graphs to illustrate bivariate correlations are displayed in Figs 4 and 5. For both plantarflexors SOL and GM, the EMG during PRE was positively correlated to the EMG in SLR and LLR, indicating the dependency of the muscles’ activation intensity during ground contact on the level of activation prior to touch-down. This was true for the drop jumps performed in the unknown condition on hard (Fig 3) as well as on soft ground (SOL: PRE and SLR (R = 0.72, P < 0.05) and PRE and MLR (R = 0.55, P < 0.05); GM: PRE and SLR (R = 0.78, P < 0.05) and PRE and MLR (R = 0.66, P < 0.05).

Strong interrelations could be established between the neuromuscular and performance parameters: PRE-EMG of the GM was positively correlated to peak force, jump height (R = 0.71, P < 0.05) and RFD, whereas the PRE-EMG of GM was negatively correlated to GCT. For the SLR, MLR and LLR, the EMG of the GM was positively correlated with peak force and RFD. In addition, a negative correlation was detected for GM EMG for the SLR, MLR and LLR and the GCT.

## Discussion

**Fig 6:**
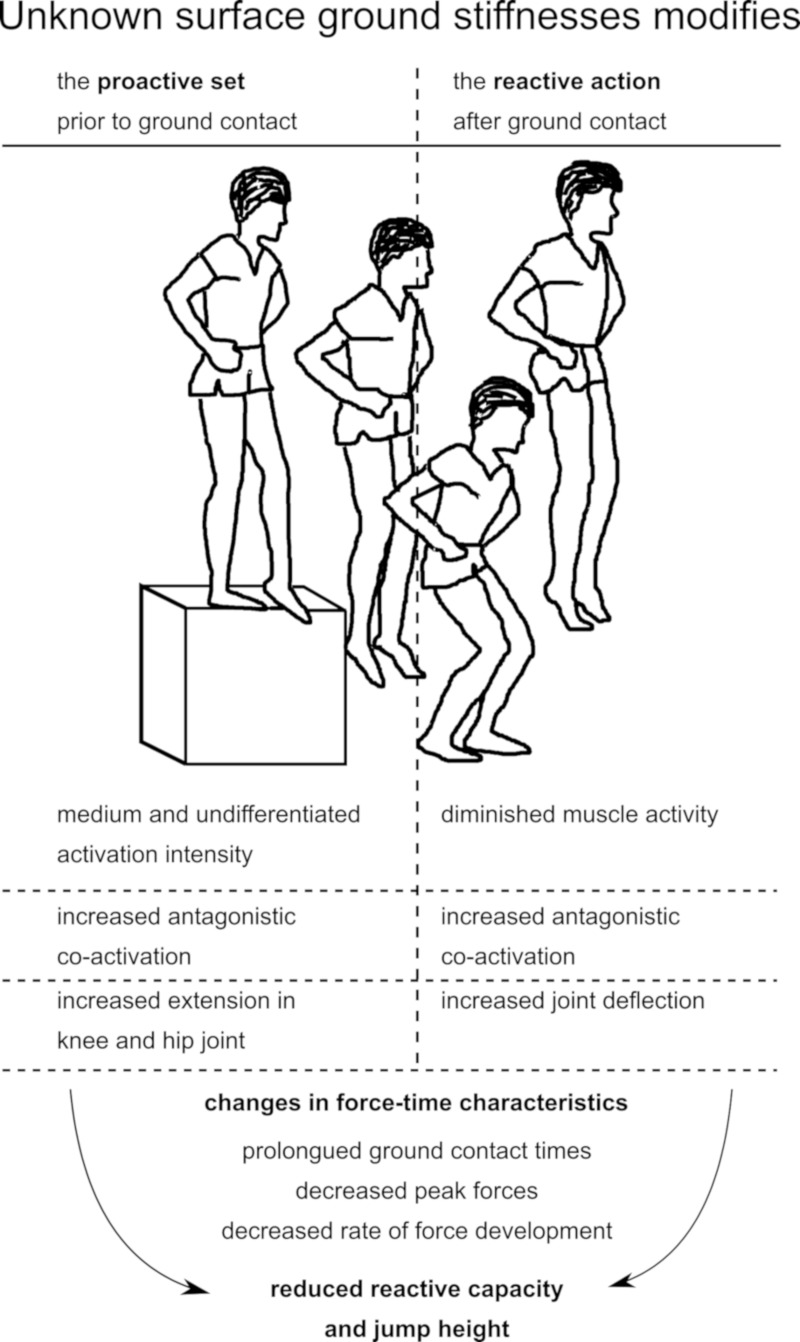
Neuromuscular and kinematic changes due to unknown ground stiffness. Changes are clustered according to the proactive (before touch-down) and reactive (after ground contact) phases of the jump.

This study investigated the effect of anticipation on forces, leg muscle activities and kinematics observed in response to changes in surface stiffness during drop jumps; the set was manipulated by providing either known or unknown SGSs. The main findings were (Fig 6): (a) muscle activation and kinematic pre-set prior to the event of GC depends on knowledge of the environmental condition. Muscular pre-activation and joint angles at initial GC did not vary in the unknown condition for both hard and soft surfaces, but varied distinctively in the known condition. (b) Regarding the reactive phase during GC, muscular activity in SOL and GM is downregulated for the eccentric phase in the unknown condition, and ROM of joints is increased in knee and hip. (c) Positive correlations of the GM EMG (PRE, SLR, MLR and LLR) with peak force and RFD underline its importance in terms of performance markers. (d) No knowledge of the upcoming condition led to a decreased performance, reflected by a decline in peak force, RFD and jump height and longer GCT.

Two aspects may be of considerable importance for the interpretation of these findings: the first one deals with proactive neuromuscular preset and its correlation to the force-time characteristics [11,19] and the second with the reactive neuromuscular control related to the biomechanical modalities and performance markers [14].

### Anticipation and proactive modulations

Proactive distinctions in the electromyographic activities prior to ground contact, reaching up to a 10% difference, were manifested between the known and unknown conditions. When ground properties were unknown, pre-activation remained unchanged regarding the soft and hard ground, indicating an undifferentiated muscular pre-set prior to ground contact, related to a declined jump performance (Fig 6). In contrast, muscular activity in the known condition was upregulated for the hard ground stiffness condition to cope with the greater expected impact and to be able to perform ballistic jumps on hard ground; and downregulated for the soft ground stiffness condition as a lower impact was expected.

First and with reference to [11,19], these outcomes underline that plantarflexor activity in the pre-activation phase is decreased when ballistic movements are performed on soft rather than hard surfaces in the known condition. This might indicate a modified feedforward activation pattern, since the central nervous system (CNS) anticipates a lower impact load at ground contact on soft and elastic surfaces [11,19,31]. In contrast, higher leg muscle activities prior to touch-down increase muscle stiffness by preparing agonistic muscles to stabilise joints protecting the musculoskeletal structures from high impact forces during ground contact on hard surfaces and to enable tendons to efficiently store elastic energy subsequently released in the concentric phase of an SSC [8,31,45]. Drop jumps are movements that are mostly pre-programmed [14], with anticipatory neuromuscular modulation occurring under the governance of supraspinal centres [22,46]. Thereby, motor control relies on an internal model within the CNS, which is supposed to predict sensorimotor consequences of the movement and the upcoming demands [46,47]. The ability to up- or downregulate muscular activity to appropriately adjust required forces to the ground stiffness is not existent in the unknown condition. Instead, the CNS chooses a medium pre-adjustment to accommodate for both forthcoming ground stiffnesses. Accordingly, knee and hip joints are slightly more extended to benefit from an early tactile detection of the SGS at touch-down. In accordance with existing motor planning theories, the motor system prepares for all potential conditions in uncertain task goals, resulting in a movement strategy which does not allow an optimized performance but provides a neuromuscular prerequisite to accommodate for the eventualities [48]. It is suggested that one of the main intentions behind this strategy is to reduce any risks of injury in uncertain movement conditions [49] while accepting the disadvantage of reduced performances (Fig 6). As indicated by the positive correlations (Fig 5), this unspecific muscular configuration prior to touch-down has an impact on the neuromuscular activities during ground contact and peak force, jump height and RFD.

Second and with reference to [19], we manifested a significantly greater antagonistic co-activation in the proactive phase in the shank musculature encompassing the ankle joint in the unknown versus known condition on hard ground. In the context of articular injury prevention, increased and congruent co-activations [50] are related to a greater joint stability and rigidity during jumping [8,51]. Co-activations are initiated by supraspinal structures [52] voluntarily aiming to secure the joint by accurately increasing the joint stiffness [53,54] and restricting the range of motion in particular trajectories in unknown, new or difficult movement conditions [53,55]. In view of the uncertainty about the required task and with reference to [19,53], our findings may indicate an injury-protective strategy to accommodate the impact forces during touch down especially within the ankle joint [19].

### Anticipation and reactive changes

Significantly reduced plantarflexor and knee extensor activities for SLR, MLR and LLR in the unknown versus known condition, reaching up to differences of 18%, were measured concomitant with changes in force-time characteristics that related to a reduced jump height and reactive performance (Fig 6). Two factors may have contributed to these neuromuscular modulations: First, the aforementioned undifferentiated proactive set during pre-activation in the unknown condition may have affected the subsequent neuromuscular activities during GC, as indicated by the positive correlations (Fig 4). However, due to its medium adjustment located in-between hard and soft ground in the unknown condition, this can only explain the diminished neuromuscular responses on hard, but not on soft ground. Second, a distinct inhibition of neuromuscular activity, coupled with modulated muscular attributes to accommodate for impact loads. Previous studies have stated that the neuromuscular activity during ground contact contributes to the muscle’s force enhancement in the eccentric phase of the movement and is of major relevance for the individuals’ force-generating capacity [22,28,56]. Interpreting the findings in view of performance and the respective significant correlations (Fig 5), it can be argued that neuromuscular inhibition in the plantarflexor muscles during GC causes a systematic magnitude-dependent shift in the force-timing characteristics of the jump and a reduced reactive capacity, reflected by a diminished peak force, RFD and jump height, while GCT was prolonged.

With an emphasis on the body kinematics during ground contact, we furthermore conclude that the decrease in the antigravity muscle’s activation intensities increased hip and knee joint deflections during ground contact [8] during the jumps with unknown SGS. Augmented joint deflections cause mechanically reduced recoil properties [14], a decline in the centre of mass and thus may have contributed to the diminished jump performance, reflected by a reduction in jump height of up to 17%. Coupled with distinctly enhanced co-activations of antagonistic muscles, which were significantly greater compared to those co-activations measured on soft ground in the known condition [19], these adaptations point towards a modified motor pattern used by the CNS when ground stiffness cannot be anticipated.

It became apparent in the scientific discourse held about drop jumps on elastic or unstable surface properties [14,19] that a shift to higher centres, reflected by an increased corticospinal excitability [14], occurs concomitant with an augmented co-activation of antagonistic musculature before and after touch-down to compensate for a decreased neuromuscular activity [14,52]. When subjects were faced with soft ground and presumably also when they were faced with an unanticipated condition, it is argued that the central nervous system compensates for the loss of stability by enhancing voluntary control during movement [14], hence impeding spinal reflex activation. It therefore can be concluded that the CNS is trying to hold onto a protective strategy reflected by the reduced agonistic muscular activation when faced with unknown ground stiffness. This seems to be supported by the enhanced antagonistic TA activation in proportion to the agonistic SOL and GM activation, expressed as the ratios TA/SOL and TA/GM. In addition, it was demonstrated that the ROM of the ankle joint in the unknown condition does not increase and hence, it could be assumed that the ankle joint might be more stable based on enhanced co-activation ratios throughout the SLR, MLR and LLR phases [49]. It seems that the thigh muscles are not that involved (P > 0.05 for BF/RF co-activation ratio), as the absorption of the main impact during uncertain situations needs to take place within the structures nearest to the impact: First, the forces are reduced (Fig 2), second, the movement is slowed down (Fig 2) and third, an increase in reciprocal inhibition caused by augmented antagonistic co-activation may have had diminished the leg extensors stretch reflex contribution mediated by the Ia afferent pathway [52,57] leading to an overall downregulation of the activation intensities during GC in favour of articular joint stabilization [55]. Consequently, our results indicate that, particularly when subjects are faced with uncertainty of the SGS with several exiting options, the maintained antagonistic activity is necessary to stabilise posture and to secure the distal joints [49,58], tolerating the disadvantage of performance decrements.

## Conclusions and functional relevance

Our knowledge about the effect of anticipation on the underlying motor control strategies that humans adopt to cope with the challenges provoked by unanticipated SGS conditions is limited. The findings of this study highlight that when ground stiffness is uncertain, the CNS is not able to adequately adjust muscular activation to accommodate a fast transition from eccentric to concentric action during drop jumps. Thereby, proactive and reactive modulations in muscle activity prior and during GC are interrelated to the force-time characteristics and height of the jumps. Further, the strategy of the CNS appears to be protective in nature through dampening impact loads via an inhibition in shank muscles as well as increased knee and hip joint deflections during GC in situations when unknown conditions are faced. In view of the interrelation of neuromuscular activity and performance, these findings underline that anticipation is a determining factor influencing timing and adjustment of motor responses to accomplish ballistic movements regarding precision and performance.

These results might also be of functional significance: Most of the prevention or rehabilitation programs that exist today focus on training in isolated tasks with correctly predicted environmental conditions. However, injuries seldomly happen through performing planned, isolated tasks in a predictable environment, and such conditions may therefore not accurately represent the dynamic environment in which most sportive activities take place [59–61]. Hence, this study may on the one side provide a basis for future research on enhancing performance in reactive activities. On the other side, further longitudinal interventions which integrate the aspect of uncertainty into training are needed that examine by which approach the CNS might learn to deal with neuromuscular modulations during unknown conditions and hence to improve motor control during those ballistic movements.
